# Double Domes of Mesoscopic Localized Anisotropic Lattice Strain in HCP–Ag_75_Al_25_ Under Uniaxial Compression

**DOI:** 10.3390/ma18071650

**Published:** 2025-04-03

**Authors:** Zhexin Sun, Mingtao Li, Nana Li, Wenge Yang

**Affiliations:** 1 Graduate School of China Academy of Engineering Physics, Beijing 100193, China; 2 Center for High Pressure Science and Technology Advanced Research, Shanghai 201203, China

**Keywords:** aggregate granular microstructure, uniaxial compression, anisotropic strain, lattice strain evolution

## Abstract

The anisotropic strain development and releasing process in materials is largely related to their intrinsic mechanical properties and mesoscale grain interactions. Uniaxial compression can induce a large amount activation energy in a system, which builds up anisotropic elastic strain. This is especially common in a hexagonal close-packed (HCP) system. Utilizing the X-ray diffraction technique, we investigated the double-dome shaped evolution of its anomalous anisotropic strain when compressing a polycrystalline HCP–silver–aluminum (Ag_75_Al_25_) alloy up to 40 GPa. Analysis of the pressure-dependent grain size showed that the anisotropic strain relaxation was accompanied with grain-size refinement. This was a strong indication of microscopic structural anisotropy impacting both the mesoscopic mechanical properties and the macroscopic fracture behavior under uniaxial compression. Our findings provide valuable novel insights for further studies on materials with anisotropic mechanical properties.

## 1. Introduction

Aggregated granular materials usually display inhomogeneous stress–strain behavior [1] from micro- to macroscopic structure scale [2]. Due to the large difference in elastic modulus along different crystallographic directions, the hexagonal close-packed (HCP) lattice has a large anisotropic lattice strain behavior [3]. In HCP materials, the anisotropic nature of the lattice strain amplifies the effect of uneven stress concentrations under pressure, which shows strong lattice-preferred orientation (LPO) [4,5] with texture evolution [6], and may further create preferential pathways for crack growth [7], leading to rapid crack propagation, which ultimately makes the material more susceptible to deformation [8,9]. When materials with anisotropic lattice stress–strain are subjected to external compression, locally dominant crystal grains can be responsible for the amplified effects of anisotropic behaviors and guide the deformation [10] until grain size homogeneously reaches the critical level [11].

HCP-structured alloys have been considered as candidates in many applications due to their excellent structure stabilities. In previous decades, binary systems such as intermetallic Ag–Al alloys have attracted much attention [12,13] due to their excellent mechanical and electrical transport properties. Compared with the disadvantages of poorer mechanical properties [14] and the high expense of pure silver counterparts, these advantages make this alloy suitable in the watchmaking industry for mechanical parts and in the semiconductor industry for bonding and the backs of solar cells. However, the anisotropic response to external stress may restrict its applicability in broader fields. High pressure has been adopted as a useful tool to study anisotropic stress–strain and fracture behavior in practical material systems. To study the anomalous anisotropic stress–strain in a material containing a mixture of coarse and fine grains, as an example of an anisotropic material, we explored the mesoscopic anisotropic mechanical properties and behaviors in an HCP–Ag_75_Al_25_ alloy under uniaxial compression. In the Ag–Al binary system, the HCP phase [12] can be formed in the composition range from 23 to 40 at.% Al. The HCP–Ag_75_Al_25_ alloy is particularly suitable for high-pressure studies due to its unique combination of moderate strength and high structural stability. Compared with other compositions, it maintains its HCP structure under high pressure, making it a promising candidate for applications requiring durability and phase stability under extreme-pressure conditions.

Previous studies into anisotropy in granular metal materials at different scales have provided a base for understanding of the link between micro- and macroscopic anisotropy. Several models and simulation methods have been proposed [15] to describe the micro–macro behavior [16,17] and properties [18] of such materials, especially HCP-structured materials [19,20]. Properties such as anisotropic elastic modulus and acoustic velocity [21,22] were studied as references and indicators for geology behaviors [23,24]. Pillar compression methods have been widely adopted for studying the anisotropy [25], as well as the conventional properties of rocks. Meanwhile, micro-pillar compression methods have also been used to explore micromechanical and macroscopic mechanical behaviors such as stress relaxation, micro-creep [26], and deformation-rate sensitivity in different anisotropic materials [27]. Previously, the majority of studies into anisotropy under compression have been focused on cubic materials [28], such as Ni, Cu, Mo, Au, Fe [29], and their cubic alloys. More recent studies have extended the range to HCP metals like Mg [30], Zr [31], HCP-Fe [32], and their alloys [33,34], and Co [35,36].

In this work, we utilized the micro-focused synchrotron X-ray diffraction technique and in situ high pressure with a diamond anvil cell (DAC) to study the evolution of localized mesoscopic anisotropic stress–strain in an HCP–Ag_75_Al_25_ alloy containing a mixture of coarse and fine grains. When the alloy was compressed under uniaxial stress with no PTM in the sample chamber, an anomalous anisotropic stress–strain diffraction pattern and its evolution with applied pressure were clearly observable.

## 2. Materials and Methods

The HCP–Ag_75_Al_25_ alloy was chosen for its moderate bulk and shear modulus and its intrinsic anisotropic HCP structure while being stable in its phase structure until reasonably high pressure, compared with a reference for HCP metal (Figure A3). The Ag_75_Al_25_ alloy was synthesized in the following steps: coarse-grained Al pellets (Aladdin, Shanghai, China) and fine-grained Ag powders (Aladdin, Shanghai, China) with purity of 99.99% were weighed in a 1:3 in molar ratio, mixed in an alumina crucible with Ar–H_2_ mixture gas protection in a glove box, and sealed under vacuum into a quartz tube. The sealed tube was heated to 720 °C over 8 h, and maintained at 720 °C for 12 h, followed by cooling over 8 h from 720 °C to room temperature without extra annealing. For uniformity, this heating–cooling process was repeated for 3 times. Finally, a coin-shaped sample was removed from the crucible, and checking with energy dispersive spectrometer (EDS) showed 74.68% and 25.35%, by atomic percentage, of Ag and Al, respectively.

**Figure 1:**
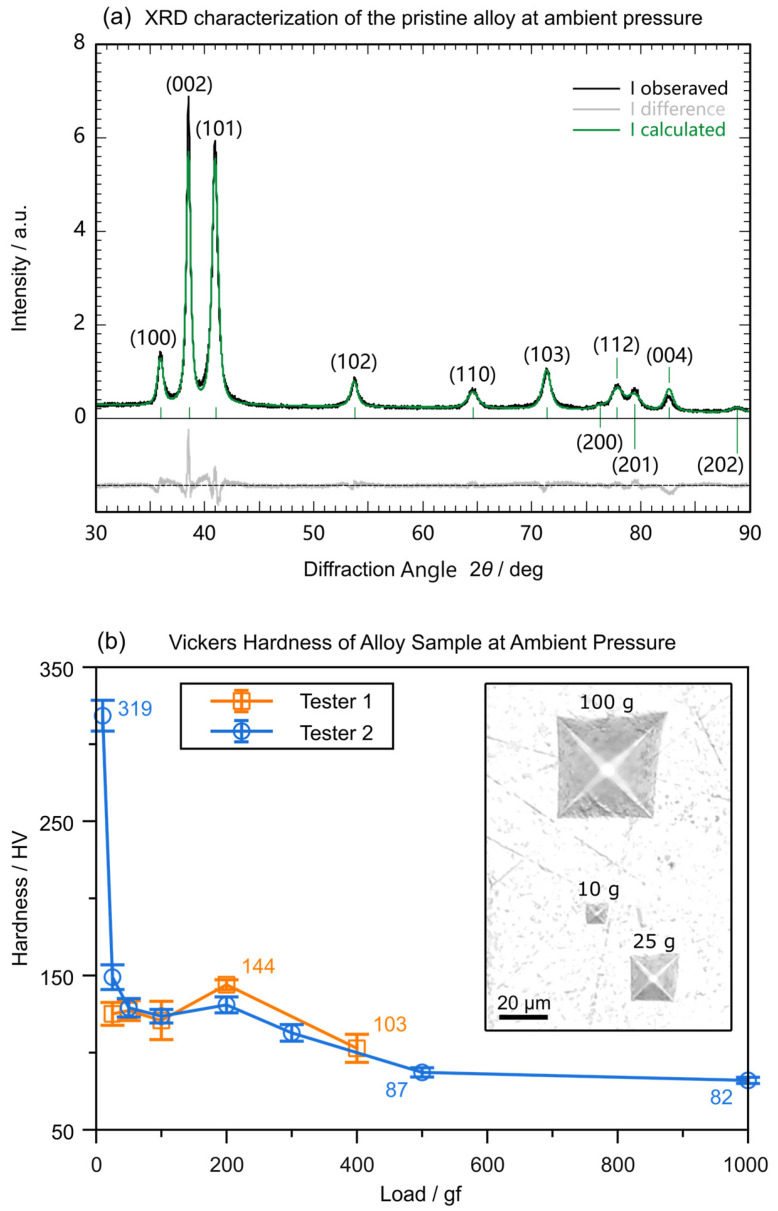
Pristine sample characterization at ambient pressure. (**a**) XRD pattern with a lab Cu-target X-ray source. (**b**) Vickers hardness testing loading up to 1 kg. The inserted picture is an optical micrograph of indentations on the polished Ag_75_Al_25_ surface.

This pristine ingot was checked with a lab X-ray diffractometer equipped with a Cu target. As shown in Figure 1a, the sample demonstrated an homogeneous solid-solution HCP structure [37]. Mechanical tests on the synthesized HCP–Ag_75_Al_25_ alloy at ambient pressure show a higher hardness and bulk modulus than pure elemental silver or aluminum with FCC structure. These properties were comparable to those of other common HCP materials like Mg or Ti alloys. A suspicious step around 200 gf in the Vickers hardness test which reproduces well across tests indicated possible grain breakage due to the release of accumulated local stress–strain at the local maximum.

**Figure 2:**
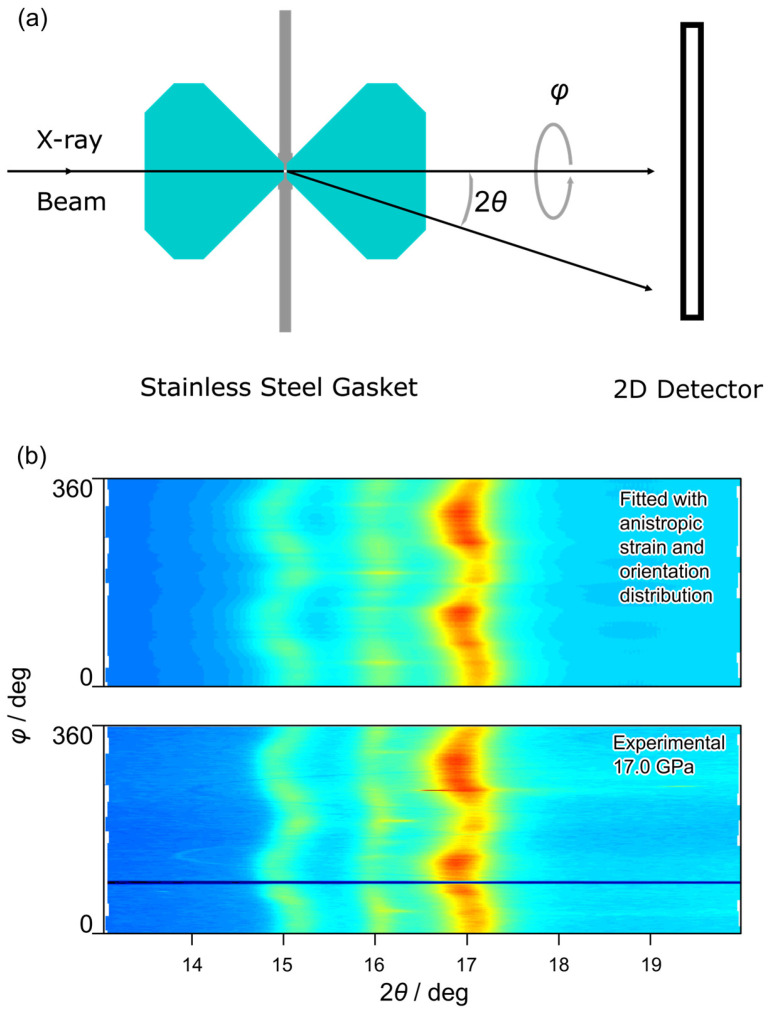
(**a**) Schematic of axial XRD experiment setup. (**b**) Contour plots of experimental and simulated XRD patterns with MAUD 2.998 of alloy specimen at 17 GPa show anisotropic strain and LPO.

High-pressure in situ XRD measurements were conducted at the Shanghai Synchrotron Radiation Facility (SSRF) (Shanghai, China). These took place at beamline 15U, with an axial diffraction geometry (Figure 2a) of DAC, and with SX165 (Rayonix, Evanston, IL, USA) as detector. The grained sample was loaded into a stainless-steel gasket sample chamber without any pressure-transmitting medium (PTM), and a micro-focused X-ray beam (about 3.5 μm×3.5 μm, FWHM) was applied with in situ non-hydrostatic high-pressure XRD measurements up to nearly 40 GPa.

It should be noted that the requirement of anisotropy in the structure of material selected, the mixed distribution of the coarse and fine grains, and the anisotropy (uniaxiality) of the applied pressure was essential for the anomalous anisotropic strain to show with axial XRD geometry in our experiment, as axial XRD would normally show no anisotropic strain pattern. A micro-focused X-ray beam was also necessary in order to probe such localized anisotropic strain. Otherwise, its effect would be averaged. For this reason, we term it as “mesoscopic”.

The two-dimensional XRD patterns were unrolled to 2θ and φ coordination with Dioptas 0.5.8 [38] for further analysis about the localized anisotropic strain. Following Singh’s method for anisotropic lattice strain analysis for material subjected to uniaxial non-hydrostatic compression, we conducted lattice strain analysis with pressures up to 40 GPa.

The initial search for interested region during XRD of the specimen was rather a random process since the design of the experiment needed both coarse grains for observable anomalous anisotropic lattice strain and fine-grained powder for a smoother diffraction ring to extract enough anisotropic data for analysis. Such regions were throughout the specimen and when an interested region with both coarse and fine grains was selected at the lowest pressure in DAC, the other measurements, at higher pressure, were kept from the same region.

The localized anomalous anisotropic pattern observed in XRD was analyzed using a conventional model for normal anisotropic lattice strain with the parameter given extra purpose to describe its anomality. When a common material is compressed by axial pressure, the dispersing of lattice strain can be described by the angle with respect to the compressing axis. Singh et al. proposed [39,40] the following equation to describe anisotropic lattice strain for material subjected to axial non-hydrostatic compression.

(1) $$\frac{d_{hkl}\left(\varphi ,p\right)-d_{hkl}\left(\varphi_{d},p\right)}{d_{hkl}\left(\varphi_{d},p\right)}=Q_{hkl}\left(p\right)\times \left[1-3\cos^{2}\left(\varphi -\varphi_{c}\right)\right]$$

where $d_{hkl}$ is the d-spacing of the (h, k,l) crystallographic plane, φ is the azimuthal angle (Figure 2a), and p is the pressure. Factor Q represents the extent of anisotropy of the strain. $\varphi_{c}$ is the azimuthal angle of compression axis, which should be 0 for conventional radial XRD [41,42] setup and was a variable in current study, as it was related to the direction of anomalous anisotropic pattern. $\varphi_{d}$ stands for the azimuth angle to give the theoretical intraplanar spacing at hydrostatic pressure, as shown in the following equation:

(2) $$3\cos^{2}\left(\varphi_{d}-\varphi_{c}\right)=1$$

## 3. Results and Discussion

**Figure 3:**
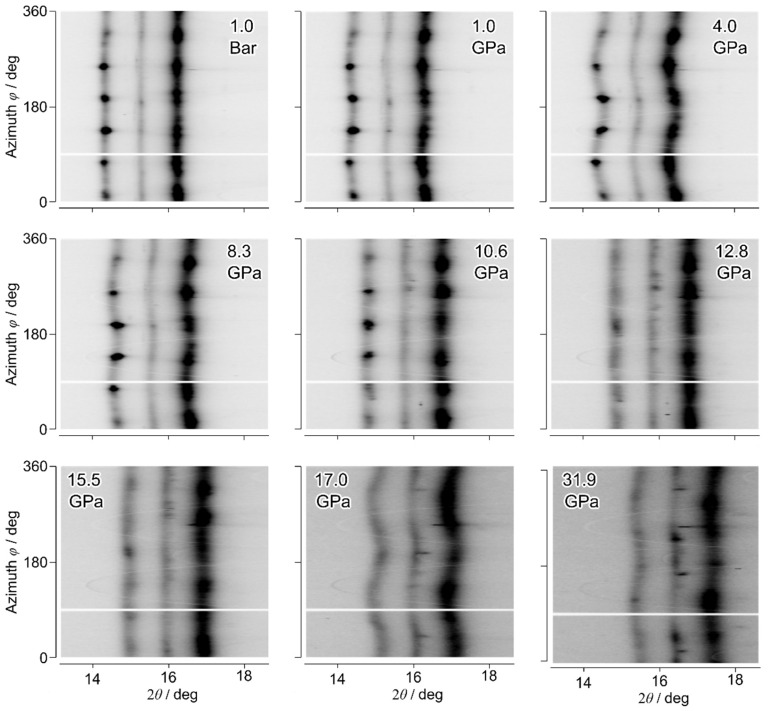
Unrolled axial XRD pattern evolution with applied pressure. Axis ticks are shared along portrait and landscape direction for a compact presentation of the pattern evolution.

Figure 3 displays the typical evolution of the axial XRD pattern in the 2θ-φ coordinate. The wavy shapes of $\left(100\right),\left(002\right),\left(101\right)$ peaks across the azimuth angle present angular-dependent lattice strain. It is evident that the amplitude of anisotropy in strain reached a maximum below 10 GPa, remained nearly zero between 10.6 and 12.8 GPa, and exhibited a second maximum around 17.0 GPa. Fitting the experimental observations with Equation (1) shows a nice adoption of this conventional model for anisotropic strains normally observed in radial XRD describing anomalous anisotropic strains in axial XRD [43], as in one example shown in Figure 2b at 17.0 GPa. Intensity variation along the azimuth direction usually indicates the present of texture. In our case, the discrete strong diffraction spots along the azimuth direction below 10.6 GPa were mainly contributed by the big grains, while the smooth diffraction intensity was scattered from fine-grain powder. After 12.8 GPa, only the fine-grain powder pattern was left, indicating a grain size diminishing process between 10.6 GPa and 12.8 GPa.

**Figure 4:**
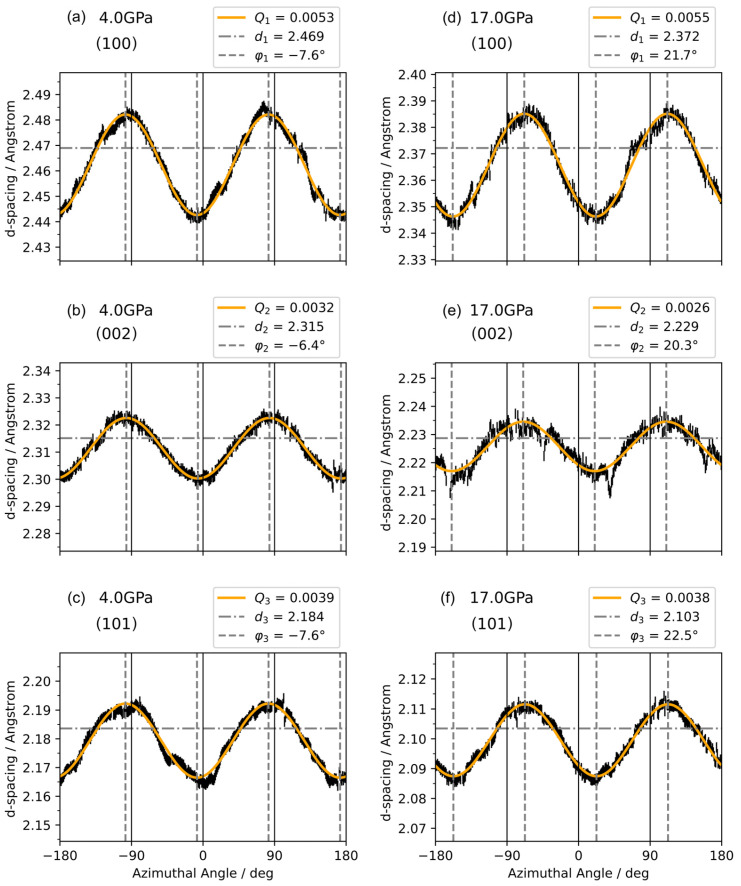
Fitting results of anomalous anisotropic strain in XRD patterns at pressures 4.0 GPa (**a**–**c**) and 17.0 GPa (**d**–**f**). Factors Q and $\varphi_{c}$ for planes $\left(100\right),\left(002\right)\;and\;\left(101\right)$ are noted as $Q_{1},Q_{2},\;{and\;Q}_{3}$ and $\varphi_{1},{\;\varphi }_{2},\;and\;\varphi_{3},$ respectively. Vertical bars are fitted peak positions at the given azimuthal angle φ.

In an isotropic structure, the lattice plane perpendicular to the compression axis should exhibit the smallest interplanar spacing, while planes parallel to the compression axis have the largest spacing. However, if the smallest interplanar spacing does not occur in the compression direction, or interplanar spacing show dispersion at the same angle with respect to the compression, then there is anomalous anisotropic lattice strain. The anomalous anisotropic strain pattern can be described with Equation (1) when it has a mechanism similar to axial compression (Figure 4).

**Figure 5:**
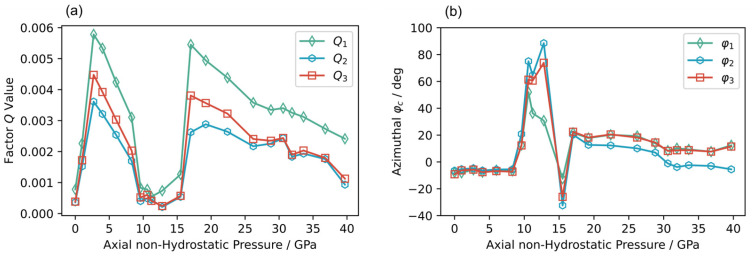
(**a**) Evolution of factors $Q_{i}$ with applied pressure. (**b**) Evolution of $\varphi_{c}$ with applied pressure.

**Figure A1:**
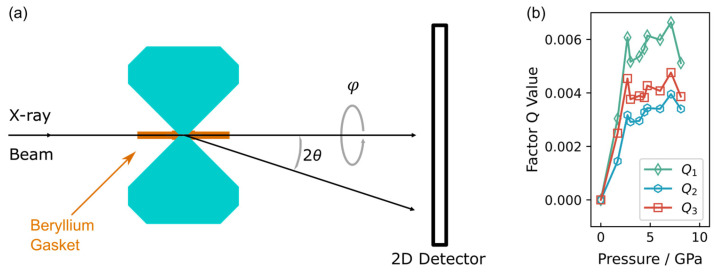
(**a**) Schematic of radial XRD setup. (**b**) Factor Q analyzed from radial XRD of the HCP–Ag_75_Al_25_ alloy.

We conducted the experimental axial XRD and fitting process up to nearly 40 GPa. The pressure-dependent $Q_{1},Q_{2}$ and $Q_{3}$ plots are summarized in Figure 5a. We can see the consistent trends for $Q_{1},{\;Q}_{2}$ and $Q_{3}$. All $Q_{i}(i=1,2,3)$ reached the first peak values at 2.7 GPa (in good agreement with radial XRD results, see Figure A1), then dropped to nearly 0 between 10 GPa and 13 GPa, followed by a rapid increase to the second peak at 17.0 GPa, and a slow decrease afterward to 40 GPa. The azimuth angle $\varphi_{c}$ also showed an anomalous change between pressures 10 and 13 GPa. For the first time, we witnessed a double dome of localized anisotropic strain response over pressure, which was largely related to mesoscopic mechanical evolution without any structural phase transition [44].

As shown in Figure 5b, $\varphi_{1},\;\varphi_{2},and\;\varphi_{3}$ were almost constant from 0 to 8 GPa, from 17 to 28 GPa, and from 32 to 40 GPa, while they changed dramatically around 10 GPa and 15 GPa and had a slight shift around 30 GPa. Analysis of $\varphi_{c}$ evolution gives more evidence of the localized anomalous anisotropic strain depending on the direction of the locally dominant grains’ micro-creep.

**Figure 6:**
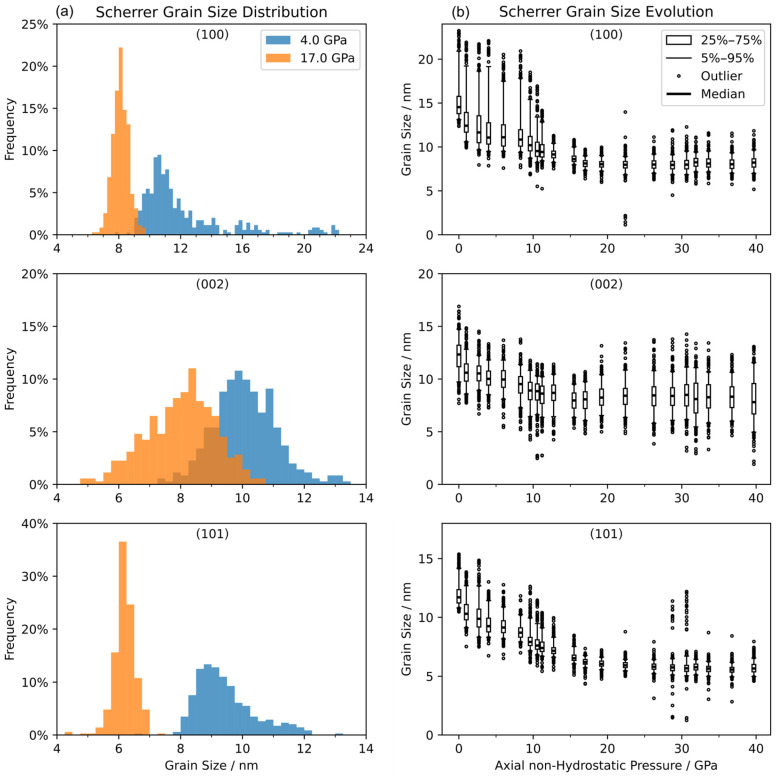
(**a**) Grain-size distribution estimated by Scherrer’s equation along the diffraction pattern ring at 4.0 GPa and 17.0 GPa. (**b**) Grain-size evolution at applied pressures.

Using the Scherrer’s equation, one can estimate the grain size from XRD peak width. We conducted grain-size analysis for all in situ high-pressure XRD measurements up to 40 GPa. In Figure 6a, we present typical grain-size distributions at two applied pressures estimated from three diffraction peaks $\left(100\right),\left(002\right),$ and $\left(101\right)$. The distributions of grain size vs. pressure are plotted in Figure 6b for all pressures measured, showing the pressure-driven grain-size evolution. The grain size had dramatically diminished between 10 and 13 GPa, where the anisotropy of anomalous strain level dropped to nearly zero, indicating the strain could be totally released during this fracturing and deformation process. After 13 GPa, the majority of the grain size was stable at sub-10 nm. It has to be mentioned that we extracted the diffraction peak width from 360 intensity vs. 2θ profiles with φ angle from 0 to 360 degrees by 1-degree intervals to avoid the artificial peak broadening from integrating anisotropic strain distribution, but diffraction spots from big grains will always contain signals from small grains because the specimen had a mixture of coarse and fine grains. This happened especially when there were more brighter spots from big grains before the dramatic fracture at measured pressures less than 15 GPa. Such underestimation of initial grain sizes does not affect the analyzed trend of grain refinement.

**Figure A2:**
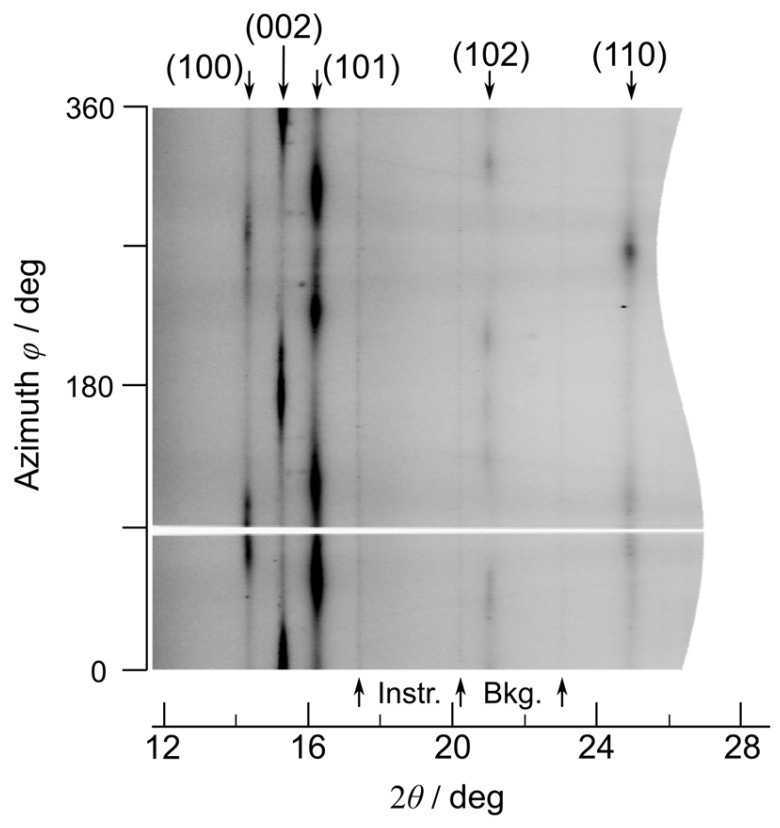
Pattern of the 10-times cycling compressed HCP–Ag_75_Al_25_ alloy specimen from radial XRD showing strong texture. Index of lattice planes (arrows at top) and instrumental backgrounds (Instr. Bkg., arrows at bottom) are marked.

**Figure A3:**
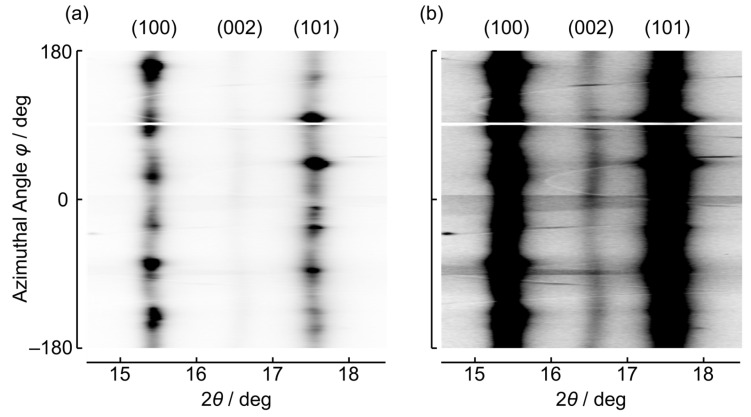
Unrolled axial XRD pattern of rhenium at 51.1 GPa. The same image is shown in (**a**,**b**), but with different applied contrast to show the bright $\left(100\right)\;and\left(101\right)$ rings and the dim $\left(002\right)$ ring.

Moreover, together with evidence of $\varphi_{c}$ evolution, the grain-size evolution (Figure 6b) also supports that while fracture of the dominant local grain happened, the localized strain changed its direction of anisotropy; otherwise, there was little change in anisotropic direction. In this specimen, grains with $\left(100\right)$ planes at exact orientation to the diffraction angle contributed the most in fracture. Grain size in the detected local area stayed almost steady after the big drop around 10 GPa, which indicates a critical grain size of this type of compression deformation that remained until the pressure that we applied (39.7 GPa). The grain size indicated by the $\left(002\right)$ plane pattern showed a different distribution of outliers above 15 GPa and the split of $\varphi_{2}$ from $\varphi_{1}$ and $\varphi_{3}$ indicates its orientation preference of micro-creep during the strain-relax and the fracture process before it, which also indicates LPO (Figure A2) in the direction of the stronger $\left(002\right)$ planes that might dominate the direction of anomalous localized anisotropy of strain in the specimen.

The relaxation behavior of the anomalous strain at the maximum critical anisotropy was consistent throughout the entire specimen. However, the most strained azimuthal angle or the pressure at which secondary anisotropy peaks appears may vary depending on the specific region where the micro-focused X-ray beam is examined. This suggests that while overall behavior was the same as the observation, reflecting the intrinsic anisotropic properties of this alloy, strain characteristics are localized and may differ slightly. As mentioned in Section 2, identifying a region of interest within the specimen is largely a random process, and it is crucial to maintain the same probed region when studying the evolution of anisotropic strain in a specific target. For this reason, we describe it as “localized”.

## 4. Conclusions

In this work, analytical results obtained from in situ high-pressure XRD on the solid solution HCP–Ag_75_Al_25_ alloy give a clear picture that localized anomalous anisotropic strain induced by axial non-hydrostatic uniaxial high pressure follows a path of rapid increase during the initial compression, reaching a critical value of anisotropy, before releasing to nearly zero through the fracture and grain-refinement process. After the sample reaches almost homogeneous grain distribution with average size below a juncture, the local strain starts to build up and reaches another maximum at critical value, followed by a slow drop upon further compression.

The moderate modulus of HCP–Ag_75_Al_25_ and its stability in structure under high pressure provided us with an excellent chance to study the evolution of such anomalous anisotropic strain in localized grains induced by the mismatch of axialities between lattice structure and uniaxial compression, with a micro-focused X-ray beam. We root the reason for the localized anomalous anisotropic strain evolution behavior upon uniaxial compression to the LPO effects brought about by the intrinsic anisotropic nature of the material’s HCP structure, which was seen in HCP–Ag_75_Al_25_ (Figure A2) and also found in other HCP-structured metals.

Though the microscopic mechanism of such localized phenomena still needs further study, by gaining a deeper understanding of behavior due to such anomalous anisotropic lattice strain evolution under uniaxial pressure through a mesoscopic view, people may fine-tune processing methods such as rolling, bending, or extruding; develop new materials with tailored properties; improve the performance of existing materials; and advance technological applications in fields like electronics, energy, and manufacturing.

## Data Availability

The original data presented in the study are openly available in FigShare at DOI: 10.6084/m9.figshare.28448858. The raw data supporting the conclusions of this article will be made available by the authors on request.

## Abbreviations

The following abbreviations are used in this manuscript:

DAC	Diamond anvil cell
EDS	Energy dispersive spectrometer
FWHM	Full width at half maximum
HCP	Hexagonal close-packed
LPO	Lattice-preferred orientation
PTM	Pressure-transmitting medium
XRD	X-ray diffraction

## Appendix A

It is quite common [45,46] to measure the normal anisotropic strain with in situ high pressure using the radial X-ray diffraction geometry, as shown in Figure A1a. An X-ray transparent beryllium gasket is used for this geometry. The direction of applied non-hydrostatic uniaxial pressure is perpendicular to the incident X-ray beam, and a panoramic DAC is used. Due to safety and health concerns related to the beryllium gasket, the highest pressure applied in this study was 8 GPa under restrict protection. The anisotropic strain factor $Q_{i}(i=1,2,3)$ at measured pressure is plotted in Figure A1b, the rapid increase of factor Q up to 2.7 GPa agrees with the measurements collected with axial XRD. After 2.7 GPa, factor Q fluctuates around its maximum, indicating the presence of fracturing.

Radial XRD can also be used to extract the LPO properties of the material. Small grains of HCP–Ag_75_Al_25_ were aggregated and then compressed, cut, and stacked. This procedure of stacking, cutting, and compressing was repeated 10 times for better exhibition of effects by LPO, using a DAC equipped with 2 mm diameter culet diamonds forming a sheet-shaped specimen of around 1.5 mm×1.5 mm. This sheet-like specimen was then cut, stacked, and compressed directly into a chamber with 300 μm diameter. Radial XRD with an X-ray beam of normal spot size at ambient pressure showed the strong texture of the LPO with diffraction intensities of $\left\langle 002\right\rangle$ distributed more around the compression direction, at φ=0° and φ=180° (Figure A2).

## Appendix B

For comparison, we conducted axial XRD measurement on a much stronger HCP metal, rhenium (99.98% commercial 0.25 mm thick foil, from Sigma-Aldrich, compressed to 30 μm thick with DAC), up to 51.1 GPa with non-hydrostatic uniaxial pressure. Anomalous anisotropic strain showed in the coarse- and fine-grained rhenium. The anomalous anisotropy was smaller than in the HCP–Ag_75_Al_25_ alloy, and no obvious evolution was observed up to 51.1 GPa. The 2θ-φ unrolled XRD pattern is presented in Figure A3 with axial non-hydrostatic pressure at the highest measured pressure of 51.1 GPa. In the pattern, it can be seen that $\varphi_{c}$ of $\left(002\right)$ differed by 51° from $\varphi_{c}$ of $\left(100\right)$ and $\left(101\right)$, which meant it could not be caused by bias from the geometry of the setup.
